# Identification of STEAP4, EPC1, and CLEC1B as non-invasive candidate biomarkers for hepatocellular carcinoma using integrated bioinformatics analysis

**DOI:** 10.1016/j.btre.2026.e00953

**Published:** 2026-02-26

**Authors:** Soheyla Khojand, Neda Zahmatkesh, Arezoo Hassani, Zahra Damerchiloo, Zahra Nikoo, Roozbeh Heidarzadehpilehrood

**Affiliations:** a Department of Biotechnology and Plant Breeding, Science and Research Branch, Islamic Azad University, Tehran, Iran; b Department of Genetics, Zanjan Branch, Islamic Azad University, Zanjan, Iran; c Department of Genetics, Faculty of Advanced Science and Technology, Tehran Medical Sciences, Islamic Azad University, Tehran, Iran; d Department of Obstetrics & Gynecology, Faculty of Medicine and Health Sciences, Universiti Putra Malaysia, 43400 Serdang, Selangor, Malaysia

**Keywords:** Non-coding RNA, Hepatocellular carcinoma, Non-invasive biomarker, Diagnostic biomarker, Prognostic biomarker, Peripheral blood mononuclear cells

## Abstract

The high global mortality of hepatocellular carcinoma (HCC) underscores the need for reliable non-invasive diagnostic biomarkers. In this study, transcriptomic analyses were performed on peripheral blood mononuclear cell (PBMC) and tumor datasets from HCC patients to identify differentially expressed genes (DEGs) using an adjusted p-value 〈 0.01 and |log2FC| 〉 1. Functional enrichment analyses revealed predominant immune-related pathways in PBMCs and metabolic pathway dysregulation in tumor tissues. Integration of PBMC and tumor profiles identified STEAP4, EPC1, CLEC1B, and LCN2 as shared DEGs. Survival analyses indicated that elevated expression of STEAP4, EPC1, and CLEC1B was associated with poorer overall survival in HCC patients. Collectively, these findings highlight consistent transcriptional alterations in PBMCs and tumor tissues and suggest that STEAP4, EPC1, and CLEC1B may serve as potential non-invasive biomarkers with diagnostic and prognostic relevance in HCC.

## Highlight

• Cytokine-related pathways dysregulated in hepatocellular carcinoma (HCC)
• *STEAP4, EPC1, CLEC1B*, and *LCN2* were identified as the shared non-invasive biomarkers.
• *Targeted miRNAs were identified, including miR-107, miR-326, GAS5, MALAT1, PRLP0P6, and SNHG1.*
• Protein-protein networks were constructed between mRNAs and miRNAs in HCC.

## 1 Introduction

Liver cancer, predominantly hepatocellular carcinoma (HCC), constitutes a major global cause of cancer-related mortality, representing 80 % to 90 % of cases [1]. It ranks sixth in morbidity and second in fatality rates among malignancies, underscoring its severe impact [2]. Early detection challenges limit surgical intervention to 10 %–20 % of HCC cases [3,4]. Without a precise treatment strategy, the median survival period for patients with advanced liver cancer is barely half a year [5,6]. Recommended systemic treatments for intermediate-to late-stage patients include chemotherapy, immunotherapy, and radiation therapy. Ambiguous clinical signs often lead to diagnoses at intermediate or advanced stages. Surgical resection or transplantation is considered in early-stage cases depending on cirrhosis status [7,8]. Chemoprevention and adjuvant therapy effectiveness are diminished in advanced HCC [9]. Alpha-fetoprotein, the sole serum biomarker for HCC, offers only around 60 % sensitivity [10]. Recognizing the urgent need for efficient and noninvasive biomarkers, PBMCs have emerged as a promising source [11]. PBMC analysis reflects host immune responses and holds promise for non-invasive early diagnosis, differentiation from healthy individuals, and prediction of tumor progression and therapeutic outcome [[12], [13], [14]]. It may also be used to predict tumor growth and therapeutic outcomes [[15], [16], [17]].

High-throughput methodologies for examining gene expression, such as next-generation sequencing (NGS) and microarray techniques, are becoming increasingly important in the field of medical oncology [18]. These technologies have diverse therapeutic applications, including molecular cancer categorization, tumor response assessment, prognosis prediction, identification of drug targets, and patient classification [19]. Notably, microarray studies have unveiled novel treatment avenues and insights into the molecular underpinnings of liver cancer, particularly in identifying the genetic abnormalities associated with HCC [20]. This study aimed to identify differentially expressed genes (DEGs) with diagnostic value by analyzing datasets from PBMC and tumors of patients with HCC, exploring the commonalities within the obtained data. Additionally, this study identified HCC-related hub genes and assessed the diagnostic significance and potential regulatory network of shared genes through overall survival analysis and the designation of non-coding RNA networks.

## 2 Material and methods

### 2.1 Microarray data

**Fig. 1:**
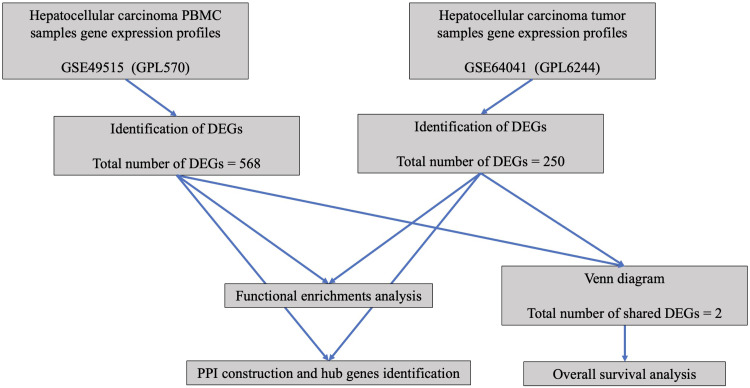
Study flowchart.

Bioinformatic analysis was performed according to the method outlined in Fig. 1. The gene expression profiles of HCC tumor tissues (GSE64041) and HCC patient PBMC (GSE49515) were collected from NCBI-GEO, an open resource of microarray datasets and next-generation sequencing. Table 1 provides the details of the datasets, including GEO accession, sample count, type of samples, and dataset platforms.

**Table 1:** HCC datasets characteristics.

GEO accession	Sample count	Type of samples	Platforms
GSE49515	20	10 HCC PBMC sample 10 Normal PBMC sample	GPL570 (Affymetrix Human Genome U133 Plus 2.0 Array)
GSE64041	120	60 HCC tumor sample 60 HCC tumor-adjacent sample	GPL6244 (Affymetrix Human Gene 1.0 ST Array [transcript (gene) version])

### 2.2 Data processing and identification of DEGs

Gene symbols were generated from probe numeric identifiers after downloading the GSE49515 and GSE64041 datasets. In the case of multiple probes associated with a single gene, an expression value deemed statistically significant was used as the gene expression value. Gene expression levels were standardized using the Affy program. Comprising CEL files (Affymetrix), the dataset files utilized for analysis comprise the raw data. The Limma program and R studio were used in the preparation and execution of the study. Linear models were employed to assess differential expression and scrutinize the experimental designs. DEGs were identified in PBMC samples from healthy controls and patients, HCC tumor samples, and HCC tumor-adjacent normal tissues using the Limma package [21] in R studio. Genes that satisfied the cut-off criteria of |log2 fold change (FC)| ≥ 1 and adjusted p-values <0.01 were chosen for further analysis. The use of a Venn diagram enabled the identification of genes that are shared between PBMC and HCC samples.

### 2.3 Functional enrichments analysis

The Kyoto Encyclopedia of Genes and Genomes (KEGG) functions as an extensive database repository that facilitates the comprehension of complex biological systems and processes by analyzing a wide range of molecular datasets generated through high-throughput experimental techniques [22]. A notable bioinformatics approach for annotating genes and analyzing their biological processes is gene ontology (GO) [23].

DEGs were subjected to biological investigation using the EnrichR online database [24]. A p-value <0.05 was considered to indicate statistical significance.

### 2.4 Protein-protein interaction (PPI) network of DEGs and Hub genes

The PPI network was constructed using STRING version 10.0, an online database of interacting genes [25]. This approach provides functional insights into disease development and progression. Interactions with a total score exceeding 0.40 were deemed statistically significant. Cytoscape, an open-source bioinformatics software (version 3.4.0), facilitated visualization of the DEG PPI network [26]. CytoHubba, a Cytoscape plugin, was used to identify the top ten hub genes based on the degree of connectivity, representing the number of direct interactions with other nodes for each protein [27].

### 2.5 Survival analysis of the shared genes

Gene Expression Profiling Interactive Analysis (GEPIA) was employed to determine the relationship between the expression levels of shared genes and the prognosis of HCC through survival analysis [28]. This database evaluates gene survival outcomes using information from The Cancer Genome Atlas (TCGA). Furthermore, data derived from the shared genes were normalized to the GAPDH gene.

### 2.6 Identification of shared gene's potential regulatory network

To elucidate the regulatory network surrounding shared genes, we utilized the miRwalk database [29] to systematically identify microRNAs (miRNAs) targeting these genes. The integration of data from multiple prediction tools within miRwalk provides comprehensive insights. miRNA involvement in liver cancer was validated using the Human MicroRNA Disease Database (HMDD) [30]. Simultaneously, the regulatory landscape was expanded by identifying liver-specific long non-coding RNAs (lncRNAs) targeting validated miRNAs, as determined using the DIANA-LncBase v3.0 database [31]. This meticulous approach resulted in a concise yet comprehensive regulatory network, revealing the intricate interplay between shared genes, miRNAs, and lncRNAs in the context of liver cancer.

### 2.7 Potential identification of shared genes co-expression modules using WGCNA analysis

The GSE64041 tissue dataset was examined in R using WGCNA package [32]. We employed R-based pipelines developed in our previous studies to generate a gene co-expression network of the shared genes for further analysis [33,34]. The threshold value was established at 50 %. To obtain the co-expressed modules, we established a minimum of 20 genes in the module and employed predefined settings for the remaining genes. Subsequently, we generated a topological overlap matrix (TOM) by employing block modular functions. We implemented a dynamic tree-cutting algorithm for clustering. We organized genes with comparable expression patterns into co-expression modules and assigned distinct colors to each module to facilitate differentiation. Genes in the co-expression module exhibited high connectivity, whereas genes within an identical module may have analogous biological functions. Subsequently, we employed the EnrichR [24] database to conduct KEGG, GO, Reactome enrichment analysis, and PPI network analysis of the co-expressed genes of the desired module.

## 3 Results

### 3.1 Identification of total and shared DEGs in HCC

**Fig. 2:**
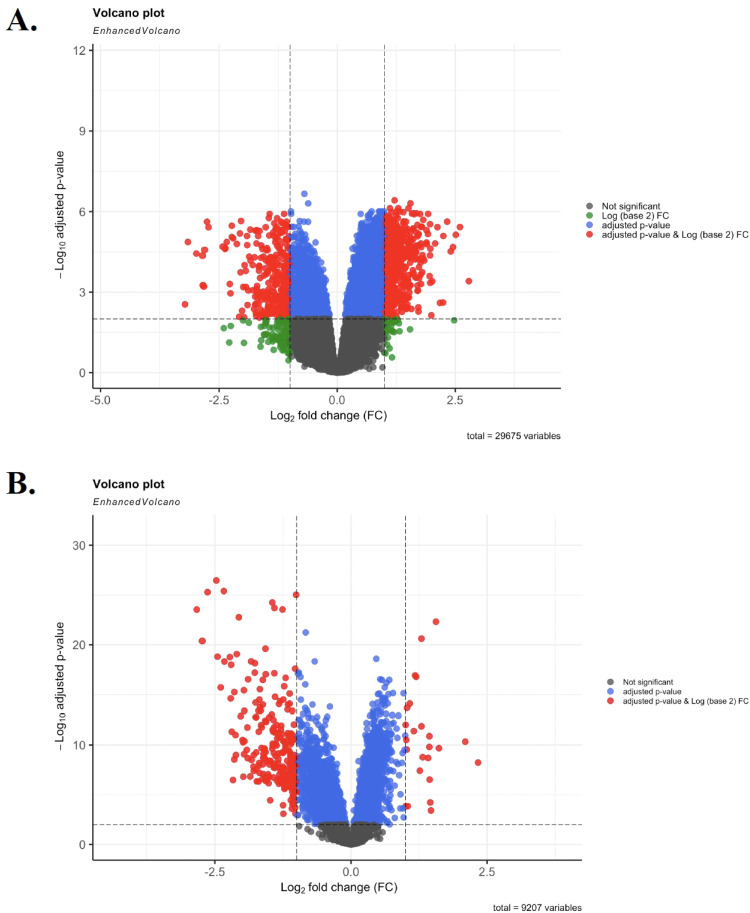
The volcano plot of the DEGs related to PBMC (A.) and tumor (B.) datasets. Screening for DEGs was done using an adjusted p-value < 0.01 and |fold change (FC)| ≥ 1.

**Fig. 3:**
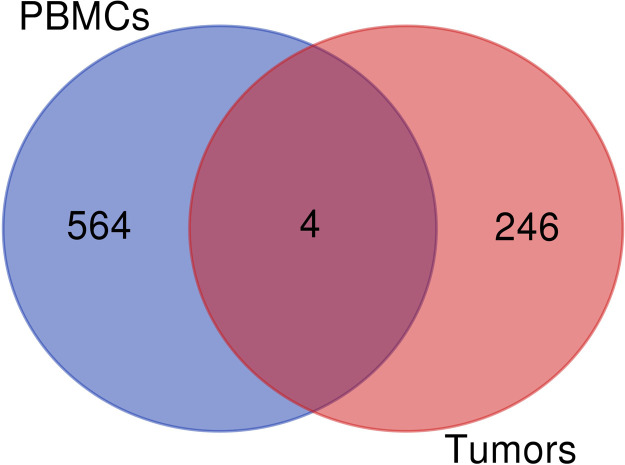
The Venn diagram between DEGs related to HCC PBMC and tumor samples. Four genes were shared between these two datasets, including *STEAP4, EPC1, LCN2*, and *CLEC1B*.

The DEGs from the two microarray datasets (GSE49515 and GSE64041) were evaluated using R software and limma package with the inclusion criteria of |log2 fold change (FC)| ≥ 1 and adjusted p-value<0.01. The rank analysis assessed the total number of DEGs, which included 2714 genes related to GSE49515 and 6666 genes related to GSE64041 (HCC tumor vs. adjacent tumor samples). A "Volcano plot" illustrating differentially expressed genes (DEGs) was generated for both datasets, depicting the relationship between p-values and fold changes (Fig. 2). Employing Venn diagram analysis (Fig. 3), a shared set of genes encompassing *STEAP4, EPC1, LCN2*, and *CLEC1B* was identified. Detailed expression data for these shared genes are shown in Table 2.

**Table 2:** The expression data of the shared genes.

Sample type	Gene symbol	Log2FC	p.value	Adj.p-value
PBMC	STEAP4	1.621	1.032E-06	0.00004
Tumor	STEAP4	−1.425	3.917E-15	3.191E-13
PBMC	EPC1	−1.162	0.0002	0.0024
Tumor	EPC1	−1.183	5.315E-08	4.728E-07
PBMC	CLEC1B	1.371	0.0008	0.0054
Tumor	CLEC1B	−2.336	8.643E-30	3.979E-26
PBMC	LCN2	1.314	0.00005	0.0007
Tumor	LCN2	1.444	3.175E-08	3.005E-07

### 3.2 Functional enrichments analysis

EnrichR was used to conduct functional and KEGG pathway enrichment analyses on DEGs to understand their biological categorization better. The top five GO biological process analyses and top five KEGG pathway enrichment analysis results are provided in Table 3, which are sorted by p-value.

**Table 3:** The functional enrichments of the HCC DEGs in PBMC and tumor samples.

Samples	Term	Library	p-value
PBMCs	cytokine-mediated signaling pathway (GO:0019,221)	GO_Biological_Process_2021	2.97E-11
	positive regulation of response to external stimulus (GO:0032,103)	GO_Biological_Process_2021	7.82E-08
	positive regulation of defense response (GO:0031,349)	GO_Biological_Process_2021	1.02E-07
	cellular response to cytokine stimulus (GO:0071,345)	GO_Biological_Process_2021	1.28E-07
	Viral protein interaction with cytokine and cytokine receptor	KEGG_2021_Human	1.34E-07
	inflammatory response (GO:0006,954)	GO_Biological_Process_2021	6.73E-07
	Cytokine-cytokine receptor interaction	KEGG_2021_Human	0.00001216
	Colorectal cancer	KEGG_2021_Human	2.9853E-05
	Influenza A	KEGG_2021_Human	3.1242E-05
	Toll-like receptor signaling pathway	KEGG_2021_Human	3.7382E-05
Tumors	epoxygenase P450 pathway (GO:0019,373)	GO_Biological_Process_2021	3.46E-11
	Retinol metabolism	KEGG_2021_Human	4.18E-11
	steroid metabolic process (GO:0008,202)	GO_Biological_Process_2021	4.38E-11
	Drug metabolism	KEGG_2021_Human	7.37E-11
	Metabolism of xenobiotics by cytochrome P450	KEGG_2021_Human	1.63E-10
	complement activation, lectin pathway (GO:0001,867)	GO_Biological_Process_2021	7.06E-10
	arachidonic acid metabolic process (GO:0019,369)	GO_Biological_Process_2021	1.93E-09
	monocarboxylic acid metabolic process (GO:0032,787)	GO_Biological_Process_2021	3.19E-09
	Chemical carcinogenesis	KEGG_2021_Human	5.66E-08
	Tryptophan metabolism	KEGG_2021_Human	7.93E-07

### 3.3 PPI analysis and hub genes identification

**Fig. 4:**
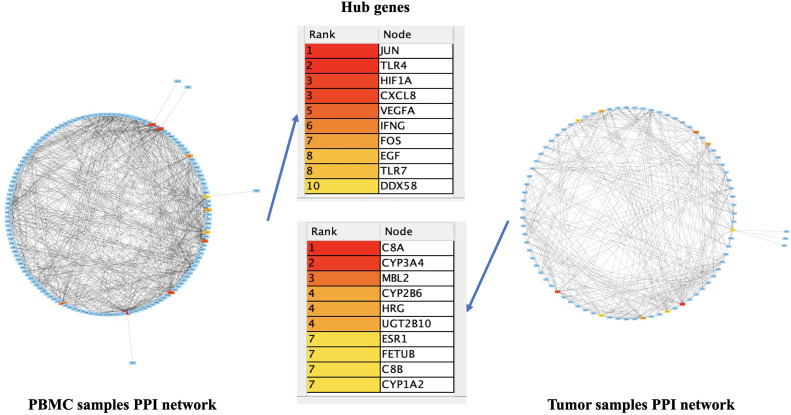
The establishment of PPI networks. Genes are represented by rectangles and the interactions between genes and proteins by lines, respectively. The hub genes associated with each network are arranged in descending order based on their degree score, ranging from one to ten.

The PPI networks were visualized using the Cytoscape STRING plugin, which included 470 nodes and 1794 edges related to the PBMC samples and 180 nodes and 664 edges related to the tumor samples. Using the Cytoscape Cytohubba plugin, the top ten hub genes were identified based on the degree of connectivity, including *PTPRE, ILF3, POLR1C, KLHDC10, USP46, NDRG1, FAAH2, FMOD, ANO5,* and *HOXC11* related to the PBMC samples and *PTPRE, ILF3, POLR1C, KLHDC10, USP46, NDRG1, FAAH2, FMOD, ANO5*, and *HOXC11* related to the tumor samples (Fig. 4).

### 3.4 Survival analysis

**Fig. 5:**
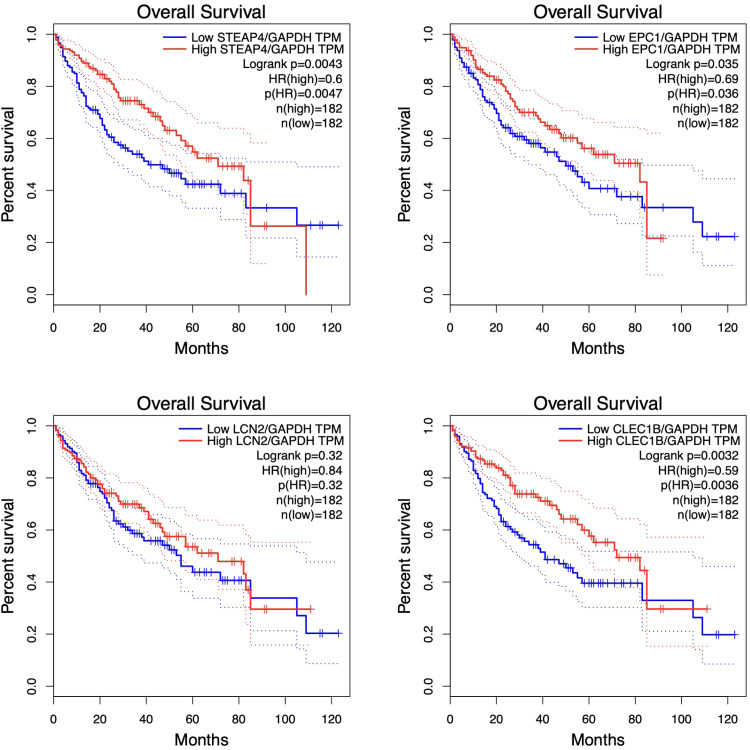
This study aims to investigate the correlation between common gene expression patterns and the clinical outcome seen in individuals diagnosed with HCC. The decreased expression levels of *STEAP4, EPC1*, and *CLEC1B* were shown to be substantially associated with worse overall survival outcomes. The *GAPDH* gene was used as a reference gene to standardize and normalize the data obtained from the targeted genes.

Significantly, the prognostic consequences of altered expression of *STEAP4, EPC1, CLEC1B*, and *LCN2* were observed via the study of overall survival and median survival time in patients. The study findings indicated that *STEAP4* (log-rank *p* = 0.0043), *EPC1* (log-rank *p* = 0.035), and *CLEC1B* (log-rank *p* = 0.0032) exhibited notable correlations with the prognosis and overall survival of HCC. However, statistical analysis did not establish a significant association between the gene expression levels of *LCN2* and patient survival (*p* < 0.05) (Fig. 5). The overall survival graph illustrates those elevated levels of *EPC1, STEAP4*, and *CLEC1B* correspond to higher survival percentages. Notably, GEPIA leverages RNA-seq data from TCGA to predict gene-specific survival rates.

### 3.5 ***STEAP4, EPC1*, and***CLEC1B***regulatory network development**

**Fig. 6:**
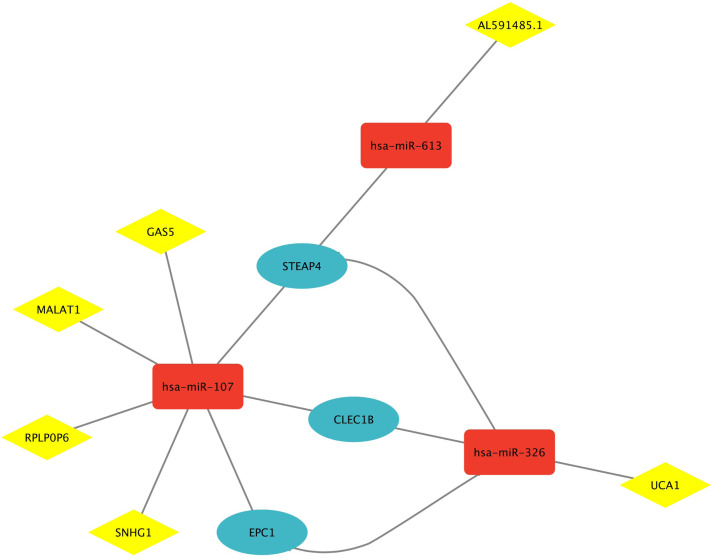
The intricate regulatory network surrounding the shared genes *STEAP4, EPC1*, and *CLEC1B* in the context of liver cancer. MiRNAs targeting these genes, obtained from the miRwalk database, underwent rigorous validation in liver cancer through HMDD. Validated miRNAs were further analyzed using the lncBASE database, revealing a detailed network involving multiple interactions. Notably, *hsa-miR-107* exhibited connections with *GAS5, MALAT1, RPLP0P6*, and *SNHG1*, while *hsa-miR-326* and *hsa-miR-613* displayed associations with *UCA1, AL591485.1*, and *STEAP4*.

After obtaining microRNAs (miRNAs) targeting *STEAP4, EPC1*, and *CLEC1B* from the miRwalk database, their validation in liver cancer was rigorously confirmed through HMDD. These validated miRNAs were subsequently input into the lncBASE database, facilitating a more in-depth exploration of the intricate regulatory networks associated with *STEAP4, EPC1*, and *CLEC1B* shared genes. The regulatory network revealed significant interactions, with *hsa-miR-107* displaying associations with various lncRNAs such as *GAS5, MALAT1, RPLP0P6*, and *SNHG1*. Additionally, *hsa-miR-326* interacted with *UCA1* and *AL591485.1*, while *hsa-miR-613* was associated with *AL591485.1* and *STEAP4*. Fig. 6 shows the complex interplay between miRNAs and lncRNAs in the context of liver cancer within the regulatory landscape of *STEAP4*-, *EPC1,* and *CLEC1B* shared genes.

### 3.6 ***STEAP4, EPC1*, and***CLEC1B***co-expressed modules network enrichments**

**Fig. 7:**
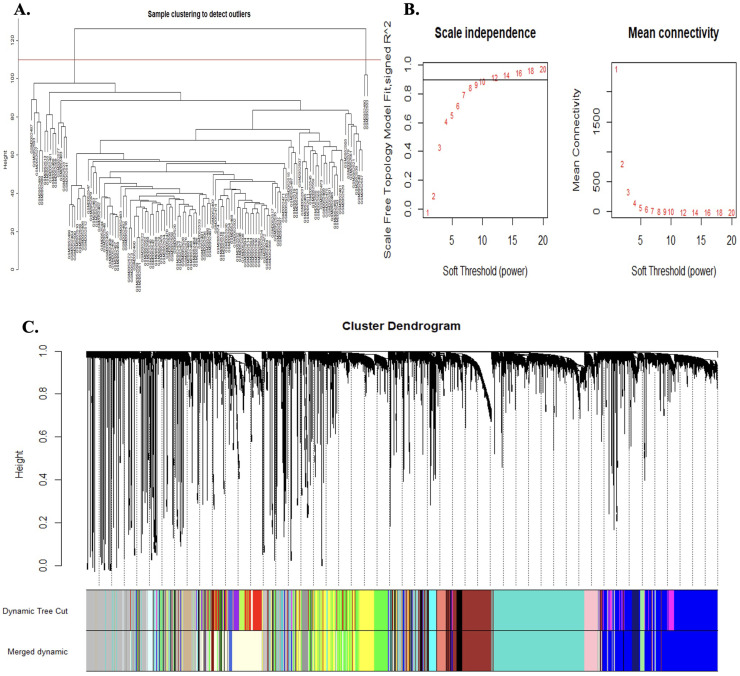
WGCNA analysis of HCC-associated DEGs. A. Detecting outliers through sample clustering, in which no outliers were identified. B. Scale independence and mean connectivity analysis, in which the soft threshold power is set at 10. C. Hierarchical cluster tree of the HCC-associated DEGs. The assigned modules and genes were represented by the color bands and the tips of the branches, respectively.

**Fig. 8:**
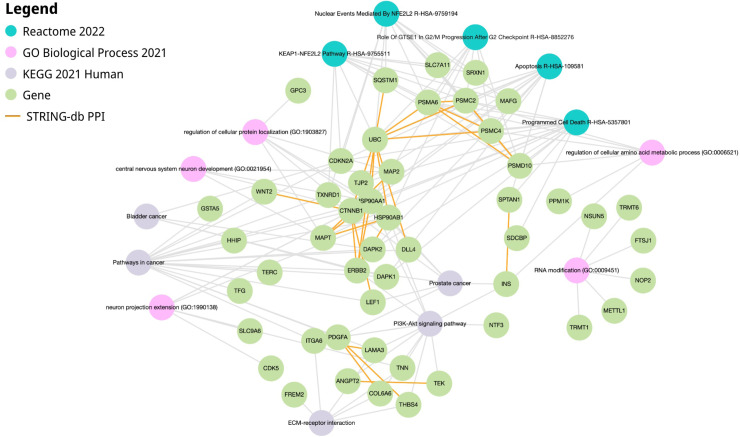
Functional enrichment of the black module co-expressed with *CLEC1B* gene*.* The analysis shows significant enrichment in processes including regulation of cellular protein localization, regulation of cellular amino acid metabolic process, and RNA modifications. Key enriched pathways identified through KEGG and Reactome include pathways in cancer, PI3K-Akt signaling pathway, bladder, and prostate cancer, apoptosis, and programmed cell death. The interaction between genes is shown using STRING-db with orange highlighted lines.

**Fig. 9:**
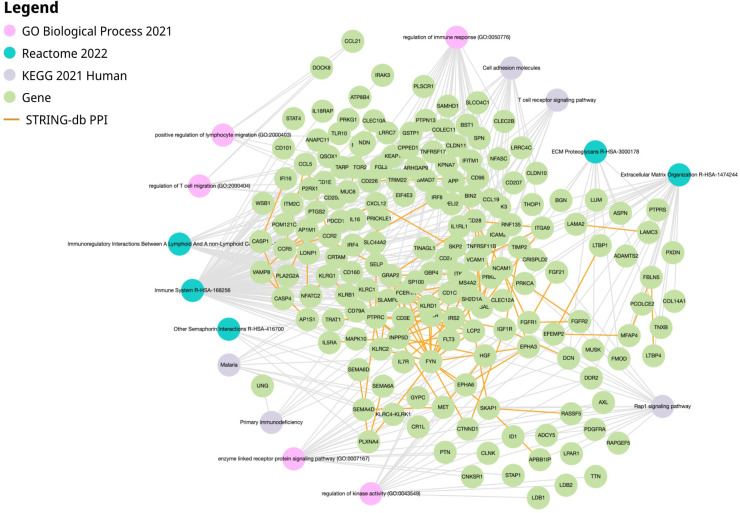
Functional enrichment of the light-yellow module co-expressed with *STEAP4* and *EPC1* genes*.* The analysis reveals significant enrichment in processes such as regulation of immune response and regulation of lymphocyte and T cell migration. Key enriched pathways identified through KEGG and Reactome include the Rap1 signaling pathway, cell adhesion molecules, T cell receptor signaling pathway, and immune receptor pathways. The interaction between genes is shown using STRING-db with orange highlighted lines.

Hierarchical clustering trees were constructed using all DEGs between tumor tissues and tumor-adjacent tissues to identify gene modules that were co-expressed with STEAP4, EPC1, and CLEC1B genes. No outliers are detected (Fig. 7A). Subsequently, co-expression analysis was conducted to establish a co-expression network. To establish a scale-free network, the soft threshold power, β, was set to 10, as shown in Fig. 7B We identified 21 co-expression modules, co-expressing the black module with CLEC1B and the light-yellow module with the STEAP4 and EPC1 genes (Fig. 7C). A total of 336 genes were identified in the black co-expression module and 881 genes were identified in the light-yellow co-expression module. GO enrichment analysis of the black module gene network co-expressed with *CLEC1B* showed enrichment in the processes of regulation of cellular protein localization, regulation of cellular amino acid metabolic processes, and RNA modifications. Enrichment of KEGG and Reactome pathways also indicated pathways in cancer, the PI3K-Akt signaling pathway, bladder and prostate cancer, apoptosis, and programmed cell death (Fig. 8). GO enrichment analysis of the light-yellow module gene network co-expressed with *STEAP4* and *EPC1* indicated enrichment in the processes of regulation of immune response and regulation of lymphocyte and T-cell migration. Enrichment of KEGG and Reactome pathways also indicated the Rap1 signaling pathway, cell adhesion molecules, T cell receptor signaling pathway, and immune receptor (Fig. 9).

## 4 Discussion

HCC, which is responsible for 90 % of liver cancer cases, remains poorly understood in terms of its molecular mechanisms [35]. Accurate diagnosis and prognosis evaluation pose significant challenges, underscoring the need for novel functional genes to enhance our understanding of HCC pathophysiology. Studies report that immune system exhaustion due to chronic hepatitis B and C virus infections contributes to the development of hepatocellular carcinoma. Although antiviral therapies increase viral suppression, dysregulation of immune system highlights the need for non-invasive biomarkers. In this context, PBMC-derived biomarkers enhance nucleic acid based immunomodulatory approaches for understanding HCC progression and identifying clinically meaningful biomarkers [36].

In this study, we performed bioinformatics analyses on differentially expressed genes associated with HCC prognosis and diagnosis, utilizing noninvasive tissue sampling to identify potential indicators for improved diagnosis and prognosis in HCC. Applying the specified criteria, 250 DEGs were identified in PBMC samples from HCC patients, while 568 DEGs were identified in HCC tumor samples, with only four common genes, *STEAP4, EPC1, LCN2*, and *CLEC1B*. Enrichment analysis of PBMC data revealed shifts in biological processes, particularly in immune response pathways, such as cytokine-mediated signaling, positive regulation of response to external stimuli, defense response, cellular response to cytokine stimulus, and inflammatory response. Changes in cytokine-related pathways, such as viral protein-cytokine receptor interactions, cytokine-cytokine receptor interactions, and cytokine-cytokine receptor interactions, were identified through KEGG pathway analysis. The crucial role of cytokines in HCC is well established, influencing tumor survival, immune evasion, and metastasis [[37], [38], [39]].

Recent integrative in-silico studies further support the robustness of network-based approaches for identifying clinically relevant genes in hepatocellular carcinoma. The current study analyzed HCC tissues and identified a set of highly co-expression hub genes, including *ASPM, RRM2, CCNB1, MKI67, HMMR*, and *EZH2*, which were associated with patient survival outcomes in and significantly upregulated in tumor samples [40]. Changes in biological process enrichment of the tumor dataset primarily involved the epoxygenase P450 pathway, steroid metabolic process, arachidonic acid metabolic process, and monocarboxylic acid metabolic process. CYP2 family members play a significant role in the CYP450 epoxidase pathway, transforming arachidonic acid (AA) to eicosatetraenoic acid (EET), which can be hydrolyzed by soluble epoxide hydrolase to less active dihydroxy-eicosatrienoic acids [41]. Certain EETs promote tumor angiogenesis through endothelial cell proliferation [42]. In contrast, tumor KEGG pathway enrichment resulted in essential metabolic pathways, including retinol metabolism, steroid metabolic process, drug metabolism, metabolism of xenobiotics by cytochrome P450, chemical carcinogenesis, and tryptophan metabolism. One of the mechanisms implicated in the progression of hepatic fibrosis is altered retinol metabolism, and enzymes involved in retinol metabolism have been linked to HCC (reviewed in [43]). The steroid metabolic pathway is also disrupted in HCC [42] because HCC patients have higher amounts of ARs in their tumor tissue and neighboring liver [44]. Liver dysfunction, evidenced by changes in metabolic homeostasis, may lead to the development of liver tumors [45].

The analysis of interactions between DEGs in PBMC and tumor samples was associated with the construction of PPI networks, from which the hub genes of these networks were also identified. Interestingly, all the roles of PBMC DEGs PPI network hub genes were identified in HCC, including *JUN* (reviewed in [46]), *TLR4* (reviewed in [47,48]), *HIF1A* (reviewed in [49]), *CXCL8* [50,51], *VEGFA* (reviewed in [52]), *IFNG* [53], *FOS* [54], *EGF* [55], *TLR7* [56], and *DDX58* [57]. Similarly, the identified tumor DEGs and PPI network hub genes have been investigated in HCC, including *C8A* [58], *CYP3A4* [59], *MBL2* [60], *CYP2B6* [61], *HRG* [62], *UGT2B10* [63], *ESR1* [64], *FETUB* [65], *C8B* [66], and *CYP1A2* [67]. Hubs are more likely to be master signaling and transcription regulators because they contribute to many connections and hold the network together [68]. Consequently, hubs have the potential to be useful therapeutic targets and biomarkers.

*STEAP4, EPC1*, and *CLEC1B* genes are potential candidates for clinical studies because they share DEGs related to PBMC and tumor samples. *CLEC1B*, classified as a C-type lectin superfamily type II transmembrane receptor, possesses one or more C-type lectin-like domains [69]. *CLEC1B* serves as a protein associated with platelets and plays a role in the activation of receptors for the snake venom toxin rhodocytin as well as its naturally occurring ligand, podoplanin. It has pivotal functions in several processes, including the separation of lymphatic and blood circulatory systems, aggregation of platelets generated by tumor cells, and modulation of immunological responses [[70], [71], [72]]. Recent research has shown that *CLEC1B* inhibits platelet aggregation and tumor metastasis, specifically in colon cancer [73]. Noteworthy findings from TCGA database align with our study, underscoring the prognostic significance of *CLEC1B* in HCC [74]. Additionally, Hu et al. identified low *CLEC1B* expression as a potential prognostic indicator in HCC, correlating it with unfavorable clinical outcomes [75]. In addition, the results of WGCNA analysis, which was conducted to identify the co-expression module with *CLEC1B*, a black module containing 336 co-expression genes, was found. In this way, the potential of the co-expression of genes with *CLEC1B* was determined through its functional enrichment indicated cancer-related biological processes pathways including the PI3K-Akt signaling pathway, apoptosis, and programmed cell death. Our study results corroborate these observations, revealing downregulated *CLEC1B* expression in HCC PBMC samples (log2FC = 1.371, adj. p-value = 0.00540), and conversely, upregulated expression in tumor samples (log2FC = −2.366, adj. p-value = 3.99E-26). In addition to the difference in expression levels of *CLEC1B* in PBMC and tumor samples added to the diagnostic value of *CLEC1B*, the overall survival analysis also supports the hypothesis that increased expression levels of *CLEC1B* in HCC are associated with increased patient survival.

One of the numerous mitochondrial proteins associated with iron metabolism that was elevated in experimental colitis is *STEAP4*, also known as the six-transmembrane epithelial antigen of prostate 4 or TNF-induced protein 9 (*TNFIAP9*). *STEAP4* is an oxidoreductase that may also serve as a metalloreductase [76]. Previous studies have established the involvement of *STEAP4* in various biological processes, including nutritional responses, inflammation, oxidative stress, and glucose and fatty acid metabolism [[77], [78], [79], [80], [81]]. STEAP4 protein expression is markedly elevated in human prostate cancer (PCa) compared to benign prostate tissue; this difference correlates with tumor grade and treatment response. Through its iron reductase activity, *STEAP4* dramatically enhances reactive oxygen species in PCa cells while simultaneously depleting NADPH levels [82]. According to other research, *STEAP4* plays a role in generating a lipopolysaccharide (LPS)- induced inflammatory milieu that promotes PCa growth [83]; Furthermore, *STEAP4* may be useful in anticipating PCa recurrence, according to Burnell et al.'s research [84]. Remarkably, STEAP4 upregulation was observed in malignant breast tissues. However, no measurable levels were found in benign breast tissues, and blocking the *STEAP4* pathway using deferiprone as an iron chelator in conjunction with the *HER2* inhibitor lapatinib resulted in a considerable decrease in cell proliferation in vitro [85]. In addition, STEAP4 upregulation was revealed in colorectal cancer (CRC) and predicted poor prognosis [86]. In contrast, recent studies have shown that *STEAP4* has a distinct role in CRC compared to *STEAPs 1–3*. The expression of *STEAP4* is not only reduced in CRC tissues compared to healthy tissues, but it also exhibits a favorable correlation with immune infiltration and markers associated with the immune system. These results imply that *STEAP4* may be a possible biomarker for estimating the degree of immune cell infiltration in CRCs [87]. *STEAP4* overexpression was linked to decreased caspase-3 activation in CRC liver metastases in an intrasplenic injection model [88]. In the present study, the results of WGCNA analysis revealed that the light-yellow module contained genes co-expressed with *STEAP4* and *EPC1.* The potential of the co-expression genes was determined through their functional enrichment and indicated the processes and pathways that can play a potential role in cancer, including processes of regulation of immune response, regulation of lymphocyte and T cell migration, Rap1 signaling pathway, cell adhesion molecules, T cell receptor signaling pathway, and immune receptors. The increased expression levels of *STEAP4* in PBMC samples (log2FC = 1.621, adj. p-value = 4.32E-5) and decreased expression levels in HCC tumor samples (log2FC = −1.425, adj. p-value = 3.19E-13) were observed. The difference in changes in the expression levels of *STEAP4* can create the potential to be identified as one of the candidate genes for HCC based on non-invasive approaches, considering that it is also known as one of the valuable diagnostic biomarkers in PCa and CRC. The overall survival analysis identified that high expression of *STEAP4* is positively associated with the percentage of patients' survival (logrank=0.0043).

Enhancer of polycomb homolog 1 (*EPC1*) serves as a protective effect against DNA damage. *EPC1* interacts with *E2F1* to suppress cell death and trigger metastasis-related gene profiles, making it a central modulator of the response to DNA damage [89]. Histone acetyltransferases (HATs), chromatin architecture, and chromatin-modifying enzymes are among the pathways known to be connected to this gene [90]. *EPC1* is a component of the NuA4 HAT complex, as proven by sophisticated investigations, and its crystal structure and molecular foundation for binding to *MBTD1* have been identified [91]. Prior investigations have identified aberrant expression of *EPC1* in endometrial stromal sarcoma [92,93]. Notably, studies involving *EPC1* knockdown revealed a consequential reduction in lung cancer cell proliferation and hindered tumor progression [94]. Additionally, *EPC1* has been associated with prognostic implications in microarray screens for nasopharyngeal cancer patients [95]. Based on the results of our study, the expression levels of *EPC1* in PBMC (log2FC = −1.162, adj. p-value = 0.00246) and tumor (log2FC = −1.183, adj. p-value = 4.73E-07) samples show a significant decrease. The exciting point of attention is the concordance and closeness of the expression levels of this gene in HCC. The results of the analysis of overall survival in HCC patients also indicated that high levels of *EPC1* were associated with an increase in the percentage of patients' survival (logrank=0.035), while the levels of this gene in tumors and PBMC were associated with a decrease. Recent study highlighted the central role of long non-coding RNAs as multi-level regulators in hepatocellular carcinoma, influencing therapeutic response through diverse molecular mechanisms, metastasis, progression and tumor initiation. Evidence indicates that dysregulated lncRNAs such as H19, GAS5, MEG3, HOTAIR, HULC and MALAT1 contribute to HCC pathogenesis by modulating epigenetic landscapes, acting as competing endogenous RNAs, and regulating key oncogenic signaling pathways including Wnt/β-catenin, PI3K/Akt/mTOR, Hippo, and JAK/STAT [96].

The regulatory network involving shared main genes, including *miR-107, miR-326, miR-613, GAS5, MALAT1, RPLP0P6*, and *SNHG1*, stands out as a critical focal point. The robust evidence of the presence of these non-coding components within the network provides compelling support for their involvement in HCC. The intricate interplay between miRNAs and lncRNAs underscores the complexity of the regulatory landscape surrounding these main shared genes. The strong validation of *miR-107* [97,98], *miR-326* [99,100], and *miR-613* [101,102], coupled with their associations with lncRNAs such as *GAS5* [103], *MALAT1* [104], and *SNHG1* [100], emphasizes the interconnected nature of these regulatory elements in the context of HCC.

In line with the emerging role of long non-coding RNAs in cancer progression, prior evidence supports the oncogenic relevance of lncRNA dysregulation across multiple tumor types [[105], [106]]. For instance, *UCA1* has been consistently reported as an upregulated lncRNA in several malignancies, including hepatocellular carcinoma, where its elevated expression correlates with advanced tumor stage, metastasis, and poor clinical outcomes. Mechanistically, *UCA1* has been implicated in modulating key oncogenic pathways such as Wnt/β-catenin and growth factor–related signaling cascades, thereby promoting tumor proliferation, invasion, and survival. These observations reinforce the concept that aberrant lncRNA-mediated regulatory networks play a critical role in hepatocarcinogenesis and support the biological relevance of the ncRNA interactions identified in the present study [107]. These findings signify the potential dysregulation of these main shared genes in HCC, indicating their relevance and significance in the molecular mechanisms underlying hepatocarcinogenesis. The robust traces of these non-coding components within the regulatory network contribute to a comprehensive understanding of the potential pathways involved in the dysregulation of key genes associated with HCC.

## 5 Limitations

Our study is limited by reliance on publicly available datasets without experimental molecular validation of gene expression or biological mechanisms. In the literature review of studies conducted on hub genes and shared genes, a study may have been conducted that was not mentioned in this study. Furthermore, further high-quality biological research with large sample sizes is required to validate our findings. Moreover, there is limitation on mechanistic insights in our study that could be acknowledge, further research should validate mechanistic importance of these biomarkers and correspondence pathways.

## 6 Future research directions

Future research may use these genes as primary data for experimental validation. The future studies could be focused on the validation of STEAP4, EPC1, and CLEC1B biomarkers and their regulatory roles in biological pathways and corresponding proteins in non-invasive manner. Alternatively, these non-invasive biomarkers could be validated as regulatory axes in the laboratory animal models, to investigate corresponding transcript factors and relate pathways in hepatocellular carcinoma progression and invasion. Moreover, future studies should leverage technologies such as CRISPR-Cas systems to functionally validate these regulatory axes in HCC models.

## 7 Conclusion

In this study, by analyzing microarray data related to PBMC and HCC tumors, we sought to identify common markers between PBMC and tumors, and four genes with altered expression, *STEAP4, EPC1, CLEC1B*, and *LCN2* were identified as common genes among DEGs. Overall survival analysis confirmed that altered expression levels of *STEAP4, EPC1*, and *CLEC1B* were significantly associated with patient survival. Functional enrichment analysis related to DEGs revealed enrichment in critical primary pathways, which in PBMC samples change in immune response processes, and in tumor samples, change in metabolic pathways that potentially affect cancer progression. In addition, the identified hub genes resulting from the PPI network also represent the known and essential genes involved in the pathogenesis and progression of HCC. These are early results, and they may be strengthened by more in vitro and in vivo research. While the potential importance of these genes at the mRNA level warrants more investigation, these results lay the groundwork for future HCC studies.

## Declarations

### Funding

The authors declare that no funds, grants, or other support were received during the preparation of this manuscript.

### Availability of data and materials

All data generated and analyzed during this study are included in the research.

## CRediT authorship contribution statement

**Soheyla Khojand:** Data curation, Conceptualization. **Neda Zahmatkesh:** Investigation, Formal analysis. **Arezoo Hassani:** Resources, Methodology. **Zahra Damerchiloo:** Software, Resources. **Zahra Nikoo:** Writing – original draft, Visualization. **Roozbeh Heidarzadehpilehrood:** Writing – review & editing, Writing – original draft, Supervision, Project administration, Methodology, Investigation, Formal analysis.

## Declaration of competing interest

The authors declare that they have no known competing financial interests or personal relationships that could have appeared to influence the work reported in this paper.

## Data Availability

All data generated and analyzed during this study are included in the research and its supplementary files.
